# Supra-systemic pulmonary hypertension after complicated percutaneous mitral balloon valvuloplasty: a case report and review of literature

**DOI:** 10.1186/s12871-021-01481-9

**Published:** 2021-10-27

**Authors:** Jose R. Navas-Blanco, Justin Miranda, Victor Gonzalez, Asif Mohammed, Oscar D. Aljure

**Affiliations:** 1 Department of Anesthesiology, Oakland University William Beaumont School of Medicine, Beaumont Hospital Royal Oak, 3601 W Mile Rd, Royal Oak, MI 48073 USA; 2 University of Miami Miller School of Medicine, Miami, FL, USA; 3 Department of Anesthesiology, Perioperative Medicine and Pain Management, University of Miami Miller School of Medicine, Jackson Memorial Hospital, Miami, FL, USA

**Keywords:** Pulmonary hypertension, Perioperative care, Heart failure, Hemodynamics

## Abstract

**Background:**

The World Symposium of Pulmonary Hypertension in 2018, updated the definition of pulmonary hypertension (PH) as mean pulmonary artery pressures (PAP) > 20 mmHg. Pulmonary venous hypertension secondary to left-heart disease, constitutes the most common cause of PH, and the determination of a co-existent pre-capillary (primary) PH becomes paramount, particularly at the moment of evaluating and managing patients with heart failure. Pulmonary artery pressures above the systemic pressures define supra-systemic PH and generally leads to frank right ventricular failure and high mortality.

**Case presentation:**

We present the perioperative management of a patient with rheumatic mitral valve disease, initially found to have severe PH due to pulmonary venous hypertension, who underwent percutaneous mitral balloon valvuloplasty complicated with mitral chordae rupture, severe mitral regurgitation and supra-systemic PH. Multiple medical therapies and an intra-aortic balloon pump were used as means of non-surgical management of this complication.

**Conclusions:**

This case report illustrates the perioperative implications of combined pre- and post-capillary PH and supra-systemic PH, as this has not been widely discussed in previous literature. A thorough literature review of the clinical characteristics of PH, methods to determine co-existent pre- and post-capillary PH components, as well as concomitant right ventricular failure is presented. Severe PH has known detrimental effects on the hemodynamic status of patients, which can ultimately lead to a decrease in effective cardiac output and poor tissue perfusion.

## Background

The World Symposium of Pulmonary Hypertension in 2018, updated the definition of pulmonary hypertension (PH) as mean pulmonary artery pressures (PAP) > 20 mmHg [1]. The presence of PH while undergoing surgery constitutes an important risk factor for increased perioperative morbidity and mortality [2, 3]. Pulmonary artery pressures above the systemic pressures define supra-systemic PH and generally leads to frank right ventricular failure and high mortality [4].

We present the case of a patient with rheumatic mitral valve disease, initially found to have severe PH secondary to left-heart disease, who underwent percutaneous mitral balloon valvuloplasty complicated with mitral chordae rupture, severe mitral regurgitation and supra-systemic PH. The patient was further managed with maximal pulmonary vasodilator therapies, inodilators and mechanical support in the form of an intra-aortic balloon pump to decrease the regurgitation fraction and relieve back-pressure into the pulmonary capillaries.

“Combined pre- and post-capillary PH” (formerly known as *“out-of-proportion”* PH), describes a group of patients with an elevated PAP, with known left-heart disease, in which the mean PAP surpasses that of the pulmonary capillary wedge pressure (PCWP) explaining the presence of a concomitant pre-capillary component in these patients [5–7]. There is currently minimal literature describing the perioperative management of patients with severe PH, which has demonstrated to have irreversible detrimental effects on these patients. This present report aims to display the clinical challenges of severe PH and combined pre- and post-capillary pulmonary hypertensive states with a thorough literature review of hemodynamic considerations and therapeutic options. Informed consent was obtained in anticipation of writing of this manuscript.

## Case presentation

A 64-year old female presented to the emergency department with sharp retrosternal pain and progressive dyspnea. She had a medical history of coronary artery disease, hypertension, atrial fibrillation, rheumatic mitral valve (MV) disease and stroke with residual left-sided hemiplegia. Transesophageal Echocardiography (TEE) revealed severe MV stenosis (MV area 0.9 cm^2^ [normal >1cm^2^]), mild mitral regurgitation (MR), no vegetations, minimal leaflet motion with pronounced thickening, extensive valvular calcification and minimal thickening of the sub-valvular apparatus (Wilkins score 14), no thrombus in the left atrial appendage and preserved left ventricular systolic function.

Left heart catheterization revealed a non-obstructive coronary artery disease. Right heart catheterization (RHC) revealed a mean PAP of 71 mmHg and a PCWP of 15 mmHg (Transpulmonary Pressure Gradient [TPG] of 51 mmHg). Given the degree of her PH and rest of her co-morbidities (Society of Thoracic Surgeons’ Surgical mortality risk 31%), a multidisciplinary team approach consisting on advanced heart failure, cardiac surgery, intensive care and cardiac anesthesia team reached the consensus that the patient was not an appropriate candidate for surgical mitral valve replacement, therefore the decision was to perform a percutaneous mitral balloon valvuloplasty (PMBV) in the cardiac catheterization under general anesthesia.

**Fig. 1:**
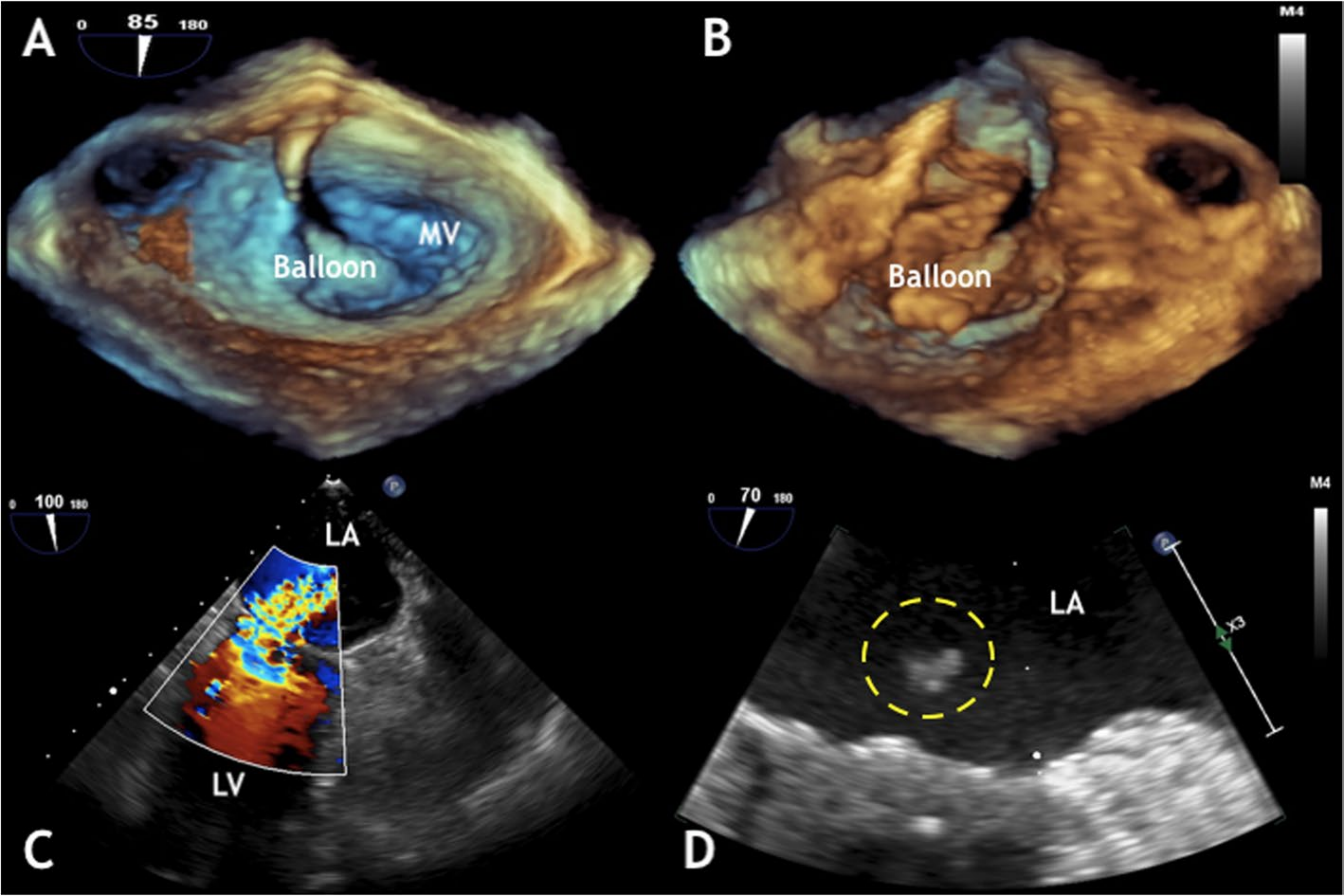
Intra-operative Transesophageal Echocardiographic imaging of the Percutaneous Mitral Balloon Valvuloplasty (PMBV). **A** Three-dimensional reconstruction demonstrating the balloon through the mitral valve (MV) coming from the left atrium, and the same view is seen from the left ventricle in **B**. **C** Immediate severe mitral regurgitation jet from the left ventricle (LV) into the left atrium (LA). **D** Piece of mitral chordae into the LA (yellow circle)

The patient was brought to the cardiac catheterization suite, underwent uneventful pre-procedure invasive lines placement (radial arterial line and right internal jugular vein introducer) and induction of general anesthesia with endotracheal tube placement. RHC demonstrated a mean PAP of 61 mmHg and a left atrial pressure of 30 mmHg. TEE revealed a severely dilated right ventricle and MV area of 0.9cm^2^. Cardiac index by Fick method was 1.97 L/min. Balloon dilation of the MV was performed, improving the effective orifice area to 1.3 cm^2^, however complicated by severe MR, secondary to a ruptured MV chordae tendineae (Fig. 1A-D). The cardiothoracic team was emergently consulted. Once again, the decision was that the patient would not tolerate undergo an emergent mitral valve replacement given her severe PH and other co-morbidities, therefore medical management would be the preferred approach in her case.

After discussion with the cardiology team, the patient was extubated and transferred to the intensive care unit (ICU) with stable hemodynamics (mean systemic arterial pressure 72 mmHg), unchanged PAP, and no signs of pulmonary edema. A pulmonary artery catheter was placed through the existing right internal jugular introducer.

**Fig. 2:**
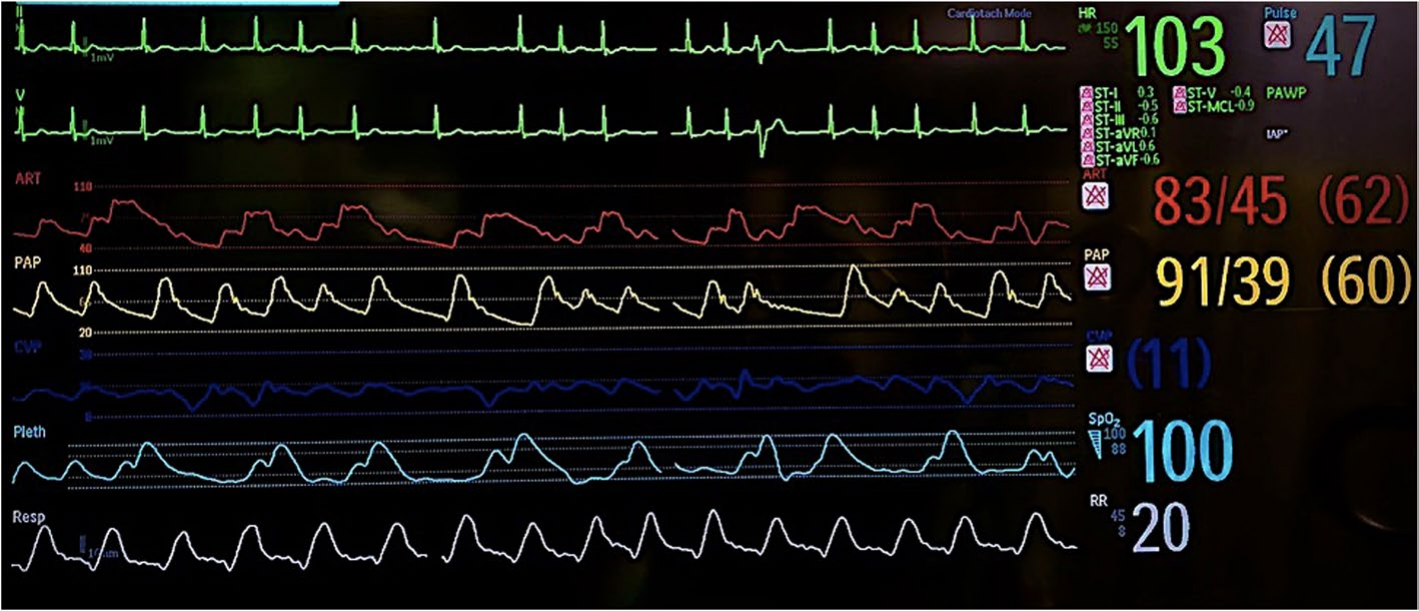
Post-procedure hemodynamics in the intensive care unit demonstrating supra-systemic pulmonary hypertension. Red waveform represents systemic arterial pressure. Yellow waveform represents pulmonary artery pressures. Blue waveform represents central venous pressure

Overnight, she developed systemic hypotension, requiring vasopressors (vasopressin), with the goal to increase the systemic pressures above the PAP, and inodilators (milrinone), with the goal to decrease the PAP. Cardiac output was borderline low at 3.8 l/minute. Mechanical support in the form of an intra-aortic balloon pump was placed to attempt decrease the MR severity and improve the cardiac output. The new severe MR further exacerbated her PH, which converted into a supra-systemic PH (Fig. 2).

Right ventricular support was initiated in the form of pulmonary vasodilators (intravenous epoprostenol − 2 ng/kg/min- and inhaled nitric oxide − 40 parts per minute- via high flow nasal cannula) and endothelin receptor antagonists (macitentan 10 mg orally, once a day). Remarkably, the patient’s pulmonary mechanics and oxygenation were preserved. At this point, the patient was managed mostly medically. Aggressive diuresis was added to the above-mentioned treatment. Eventually, given a recalcitrant response to maximal medical therapy, the patient and her family opted for hospice care and comfort measures.

## Discussion and conclusions

We describe the perioperative care of a patient with severe PH associated to a rheumatic mitral stenosis who developed supra-systemic PH after a complicated PMBV. The American College of Cardiology recommends PMBV approach for patients with severe mitral stenosis found to be high risk for surgery due to co-morbidities and with favorable mitral valve anatomy. Severe mitral regurgitation after PMBV is relatively rare (1.4–9.4%), with most of the mechanisms associated to chordae tendinea rupture or damage to the sub-valvular apparatus [8].

**Fig. 3:**
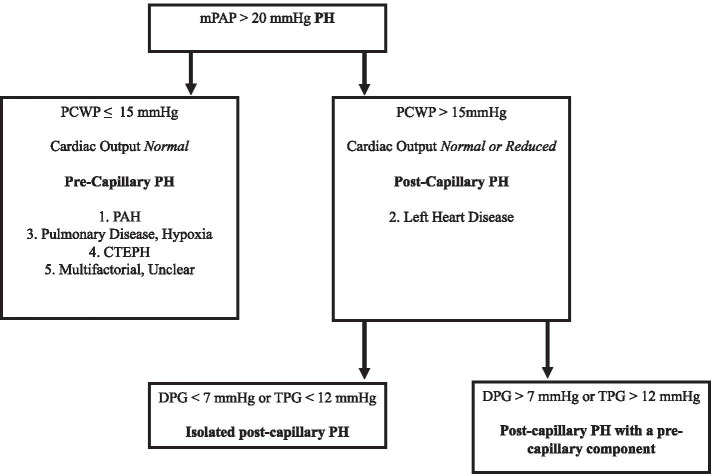
European Society of Cardiology and European Respiratory Society Guidelines for Diagnosis of Pulmonary Hypertension (PH). PAH = Pulmonary Arterial Hypertension; PCWP = Pulmonary Capillary Wedge Pressure; CTEPH = Chronic Thromboembolic Pulmonary Hypertension; DPG = Diastolic Pulmonary Gradient; TPG = Transpulmonary Gradient

The World Health Organization classifies clinical instances of PH in five categories as stated in Fig. 3 [5]. Patients in clinical group 2 – the most common cause of PH –, are defined as mean PAP > 20 mmHg and a PCWP (a surrogate of left atrial pressure) > 15 mmHg, with a normal or reduced cardiac index [9]. These patients have an initial intact intrinsic pulmonary vascular tone and the elevated PAP is a consequence of pressure build-up coming from the left-sided chambers of the heart due to multiple etiologies [10].

In the case presented, the patient initially had a group 2 PH, with an added pre- and post-capillary PH component, based on a transpulmonary pressure gradient of 51 mmHg and a decreased cardiac index, which prompted the multidisciplinary team to pursue to correct the left-heart pathology (mitral stenosis) as the potential root of the problem. At this point, the decision was to proceed with the PMBV, with the unfortunate shortcoming of the mitral chordae rupture which led to severe MR and supra-systemic PH. Given the complexity of perioperative PH, its diagnosis and management, a thorough review becomes necessary to highlight the following educational points extracted from this case.

### Pathophysiology and terminology

The underlying mechanism associated to PH due to left-heart disease includes a “passive” increase in the mean PAP due to loss left atrial compliance and diastolic dysfunction, which leads venous congestion, structural changes in the pulmonary arterioles due to endothelial dysfunction, vasoconstriction, vascular remodeling and blunted response to vasodilators [10]. Eventually, chronic right ventricular pressure overload resulting from PH ultimately leads to cavity dilation and contractile dysfunction. Patients with “isolated post-capillary PH” may have a persistent elevated PAP even after the underlying left-ventricular problem is controlled, as reversing the above-mentioned pathophysiologic mechanisms requires time [6, 11].

Conversely, Group 2 patients may also have a co-existent pre-capillary (primary) PH component, which significantly affects their prognosis. These patients often have a different clinical history and their management is as expected different, since controlling their left-heart disease will not relieve their PH. Based on this mechanism, multiple terms have been presented to classify this population of patients, including “passive” versus “active” PH, as well as “out-of-proportion” PH [12]. The most recent update of the European Society of Cardiology and the European Respiratory Society guidelines on PH, adopted the classification of “isolated post-capillary PH” (as a substitute of “passive” PH) and “combined pre- and post- capillary PH” (as a substitute of “active” PH and “out-of-proportion” PH), based on the concepts of Transpulmonary Pressure Gradient (TPG) and Diastolic Pressure Gradient (DPG) [7].

### Parameters to define combined pre- and post-capillary pulmonary hypertension

TPG is defined as the difference between the mean PAP and the PCWP. A large retrospective study performed by Gerges et al. demonstrated that in patients with combined pre- and post-capillary PH a cut-off value for TPG of > 12 mmHg was associated with a worse median survival at 78 months. However, TPG was later found to be less sensitive to define a combined PH component in patients with left-heart disease, given its dependency to loading conditions, cardiac output, pulmonary flow and resistance as well as left atrial pressure [7]. In contrast, the concept of DPG, which involves the difference between diastolic PAP and PCWP, is less affected by any of the prior factors altering the TPG. The cut-off recommended by the European Society of Cardiology and the European Respiratory Society guidelines for DPG is > 7 mmHg to define patients with combined pre- and post-capillary PH (Fig. 3) [13].

### Right ventricular failure and pulmonary artery pressures

Right Ventricular Failure (RVF) represents a highly complex clinical entity without a universal definition and without a single hemodynamic parameter accurate enough to predict or classify the degree of RVF. Invasive techniques in the form of a right heart catheterization provides useful information that allows the clinician to understand the function of the RV and echocardiography provides also helpful parameters for qualitative and quantitative assessment [4, 14–16]. RVF has even higher interest in the presence of high PAP, and more so, in patients with severely decreased left ventricular function, as a poor RV performance is associated with poor outcomes in this population of patients. The presence of elevated CVP values (> 18 mmHg) with a depressed cardiac index (< 2 L/min/m^2^) in the absence of an elevated PCWP (> 18 mmHg), aids the clinician to determine the presence of RVF, although quantifying the severity of this failure is usually made clinically, based on the response to inotropes, pulmonary vasodilators and fluid responsiveness [14].

Determination of RVF in patients with isolated pre-capillary PH and combined pre- and post-capillary PH is paramount since ultimately decides mortality in this group of patients. RVF often requires inotropic support, fluid restriction, afterload reduction agents, diuretics and selective pulmonary vasodilators (e.g. epoprostenol, nitric oxide) or pulmonary endothelin receptor blockers (e.g. macitentan, bosentan), all of these in an intensive care unit setting. Mechanical support in the form of right ventricular assist device (RVAD) may also be required in these patients [6, 13, 17, 18].

The use of intra-aortic balloon pump (IABP) as a mechanical support for patients with RVF remains debated and clinical data evaluating the use of such device for this purpose is limited. Nevertheless, IABP is used as a mean to improve coronary perfusion pressure for both the right and left ventricle, as well as to decrease aortic impedance, promoting antegrade flow, decreasing the regurgitant fraction through the mitral valve and relieving the pressure build-up back into the pulmonary artery tree [18, 19].

### Pulmonary artery Pulsatility index

Multiple hemodynamic parameters have been discussed with the purpose to diagnose RVF, especially in the setting of PH where elevated afterload in the pulmonary tree poses an increased risk for RVF and this is the rationale for the introduction of the Pulmonary Artery Pulsatility Index (PAPi) [20]. PAPi is a hemodynamic parameter derived from right atrial and pulmonary artery pressures, as following:

$$PAPi=\frac{PAP_{systolic}-{PAP}_{diastolic}}{Central\ Venous\ Pressure}$$

The fundamental principle of PAPi as an indicator of right heart function is founded on the systolic PAP as an indirect indicator of contractility of the right ventricle against afterload and a high central venous pressure (surrogate for right atrial pressure) as a sign of failing right ventricle [21]. PAPi was initially described to assess the risk of RVF after acute myocardial infarction and to determine the need for such patients for implantation of a RVAD, but given its satisfactory predictive properties, its use has expanded to other clinical scenarios including post-myocardial infarction, post-cardiotomy, post-left ventricular assist device implantation (LVAD) and as a predictor of graft function after heart transplantation [17, 20, 22]. Kang et al. in a retrospective analysis of 85 patients who underwent LVAD implantation, found that a higher PAPi was associated with a reduced risk for RVAD placement in these patients. In this study, optimal sensitivity and specificity was achieved when using a PAPi threshold of 2.0 [17]. Assessment of PAPi becomes trivial given the presence of supra-systemic PH, and this may represent a potential disadvantage of this hemodynamic measurement.

## Conclusion

The present case exemplifies the complexity of the management of patients with combined pre- and post-capillary PH. This population of patients poses an increased anesthetic and intensive care risk, given the delicate balance to optimize right ventricular function to promote forward flow and effective cardiac output in the presence of a high afterload in the pulmonary tree. Overall, maximal hemodynamic monitoring is required in order to first classify the cause of the PH in the patient, the presence of an isolated versus a combined PH, and based on this, tailor a specific therapy (surgical versus medical). Final clinical conduct should be made in a multidisciplinary fashion in order to reach the most adequate consensus to increase the survivability in these patients.

## Data Availability

Not applicable.

## Abbreviations

PH: Pulmonary Hypertension
PAP: Pulmonary Artery Pressures
PCWP: Pulmonary Capillary Wedge Pressure
ED: Emergency Department
MV: Mitral Valve
TEE: Transesophageal Echocardiography
RHC: Right Heart Catheterization
TPG: Transpulmonary Pressure Gradient
MVR: Mitral Valve Replacement
PMBV: Percutaneous Mitral Balloon Valvuloplasty
ICU: Intensive Care Unit
CVP: Central Venous Pressure
DPG: Diastolic Pressure Gradient
RVF: Right Ventricular Failure
RVAD: Right Ventricular Assist Device
IABP: Intra-Aortic Balloon Pump
PAPi: Pulmonary Artery Pulsatility Index
LVAD: Left Ventricular Assist Device
